# Supplementary figures and images for: Noninvasive analysis of metabolic changes following nutrient input into diverse fish species, as investigated by metabolic and microbial profiling approaches
Source: PeerJ. 2014 Oct 28;2:e550. doi: 10.7717/peerj.550 (PMC4217172; doi:10.7717/peerj.550)

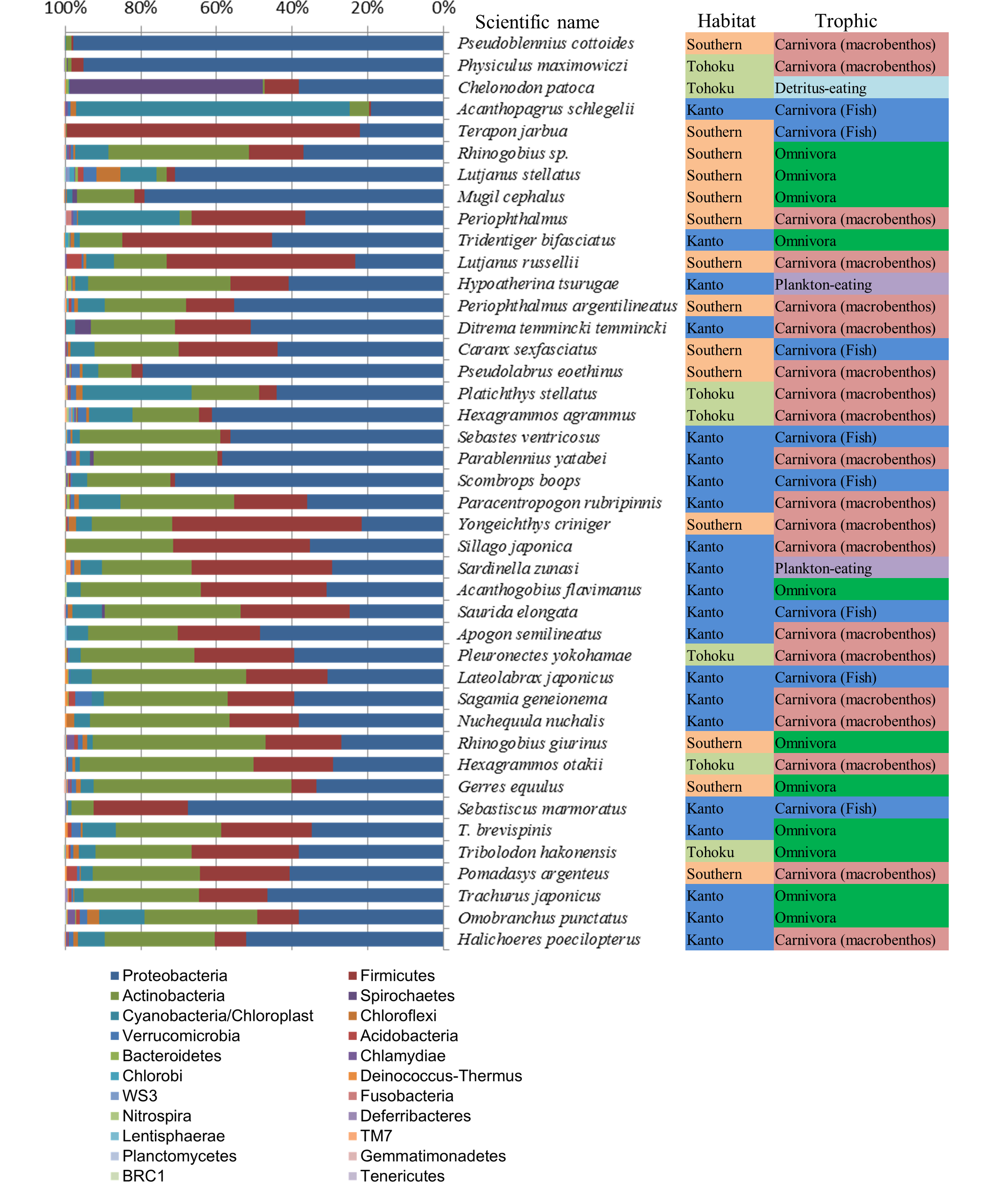

Supplement: Figure S1 — Bacterial species from 42 fish gut communities classified into the phylum level. Bar graphs for each library represent the percentage of each phylum. Scientific names of the fish hosts are listed along the horizontal axis. Habitat and food habits of these fish are described in Table S1. [file peerj-02-550-s001.png]

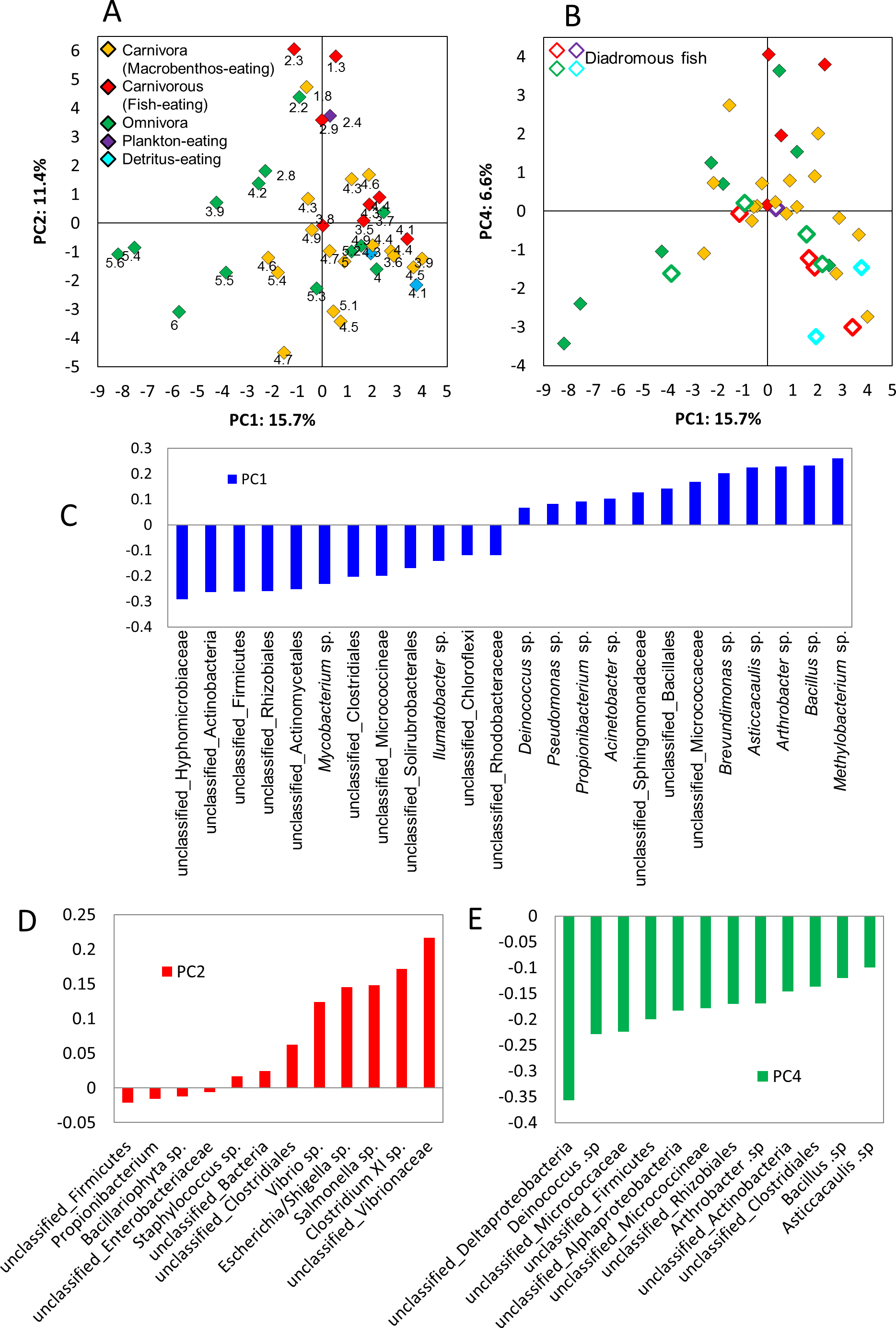

Supplement: Figure S2 — Microbiota profiles from fish guts evaluated by PCA (n = 41, k = 52, R2X = 0.154, R2Y = 0.114, Q2 = 0.059). (A) PCA score plot based on PC1 and PC2. The numbers on PCA denote the values of species diversity calculated by the Shannon diversity index. (B) PCA score plot based on PC1 and PC4. (C, D, E) PCA loading plots for PC1 (C), PC2 (D), and PC4 (E) of top 12 positives (C, D, E) and negatives (C). [file peerj-02-550-s002.png]

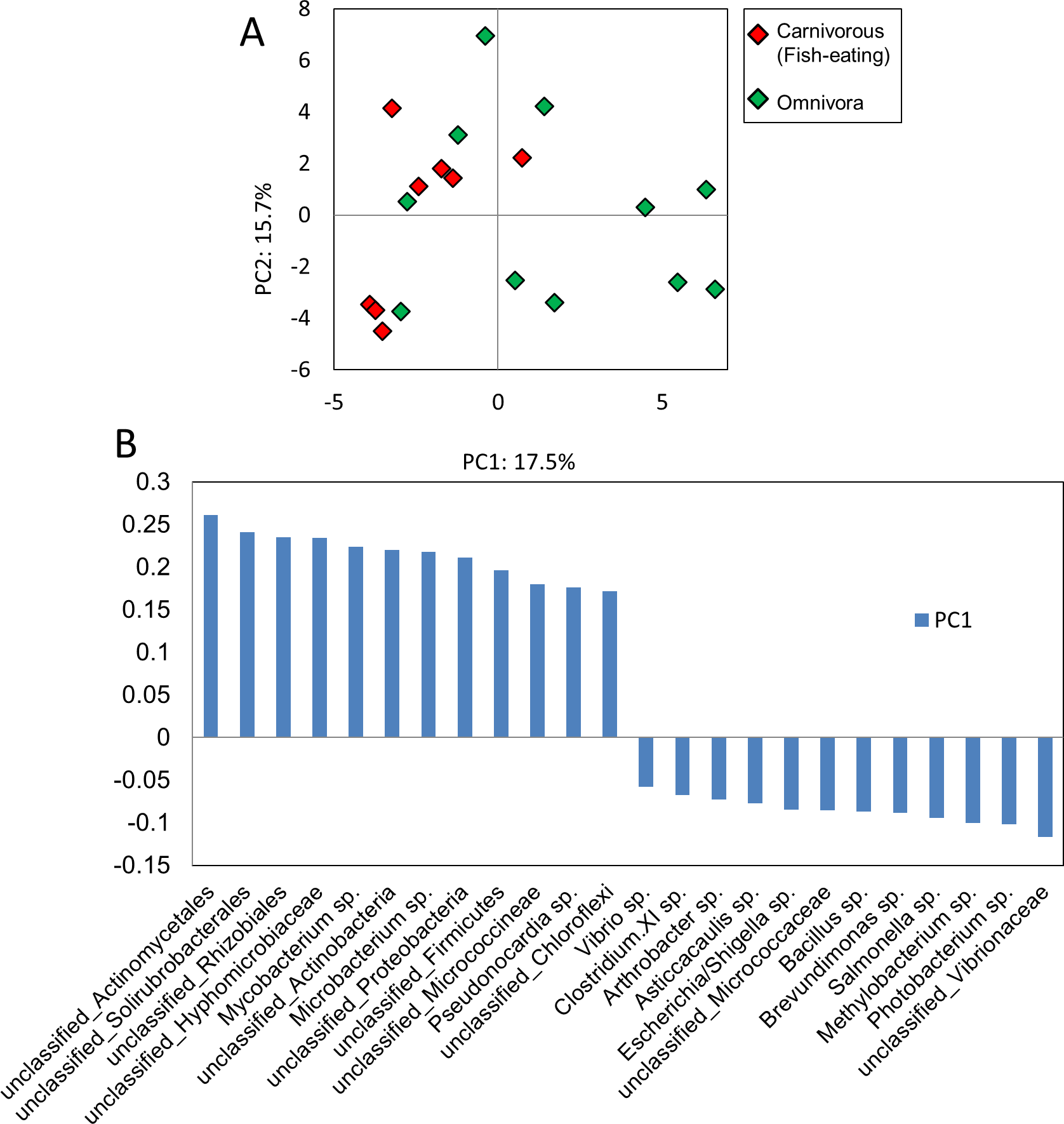

Supplement: Figure S3 — Microbiota profiles from fish guts evaluated by PCA (n = 19, k = 69, R2X = 0.175, R2Y = 0.158, Q2 = 0.018) for comparison between fish eaters and Omnivora. (A) PCA score plot based on PC1 and PC2. Symbols representing individual communities are colored by Fish eater (red) and Omnivora (green). (B) PCA loading plots for PC1. [file peerj-02-550-s003.png]

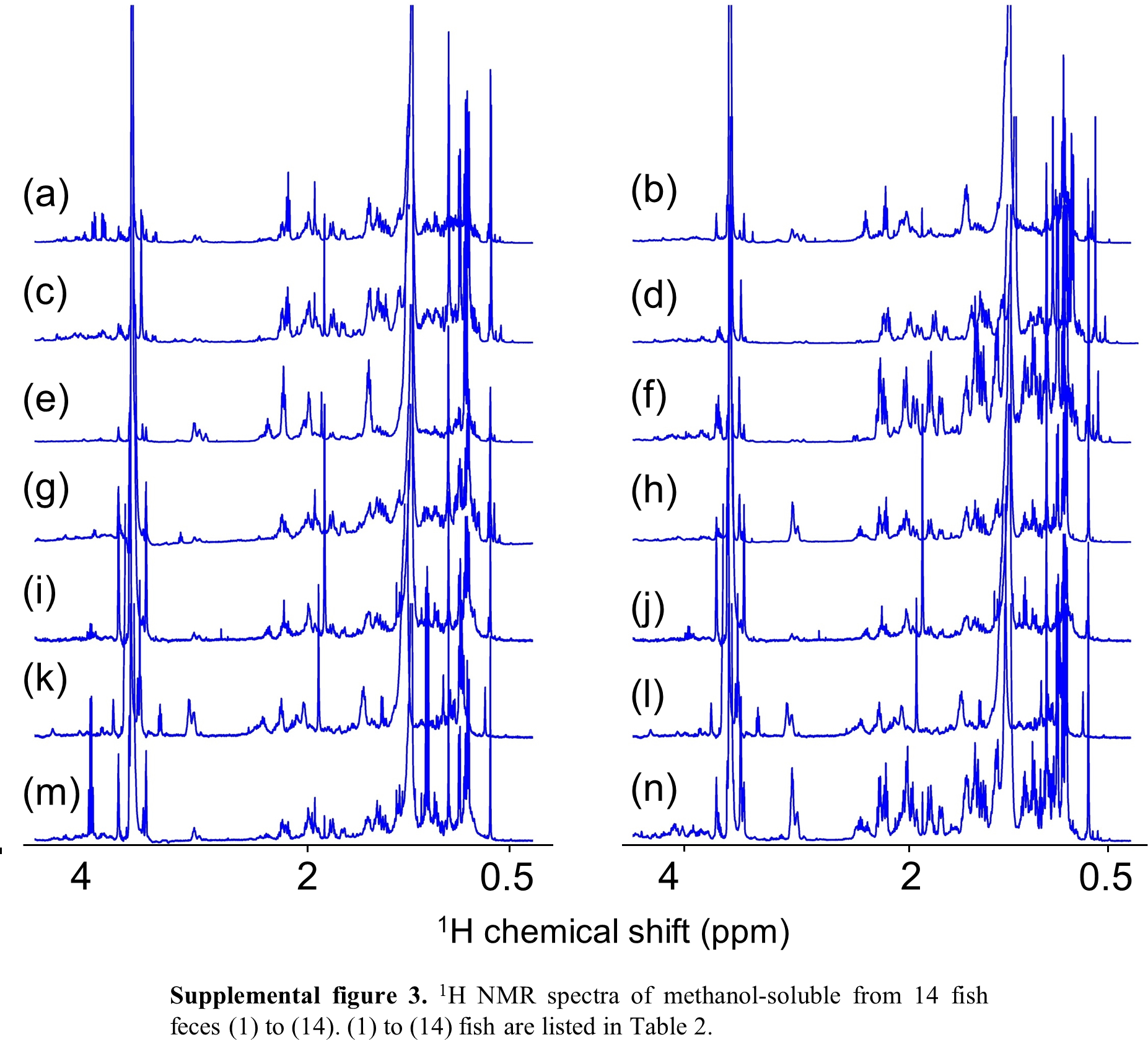

Supplement: Figure S4 — 1H NMR spectra of methanol-soluble fraction from 14 fish feces (a) to (n). (a) to (n) fish are listed in Table S3. [file peerj-02-550-s004.png]

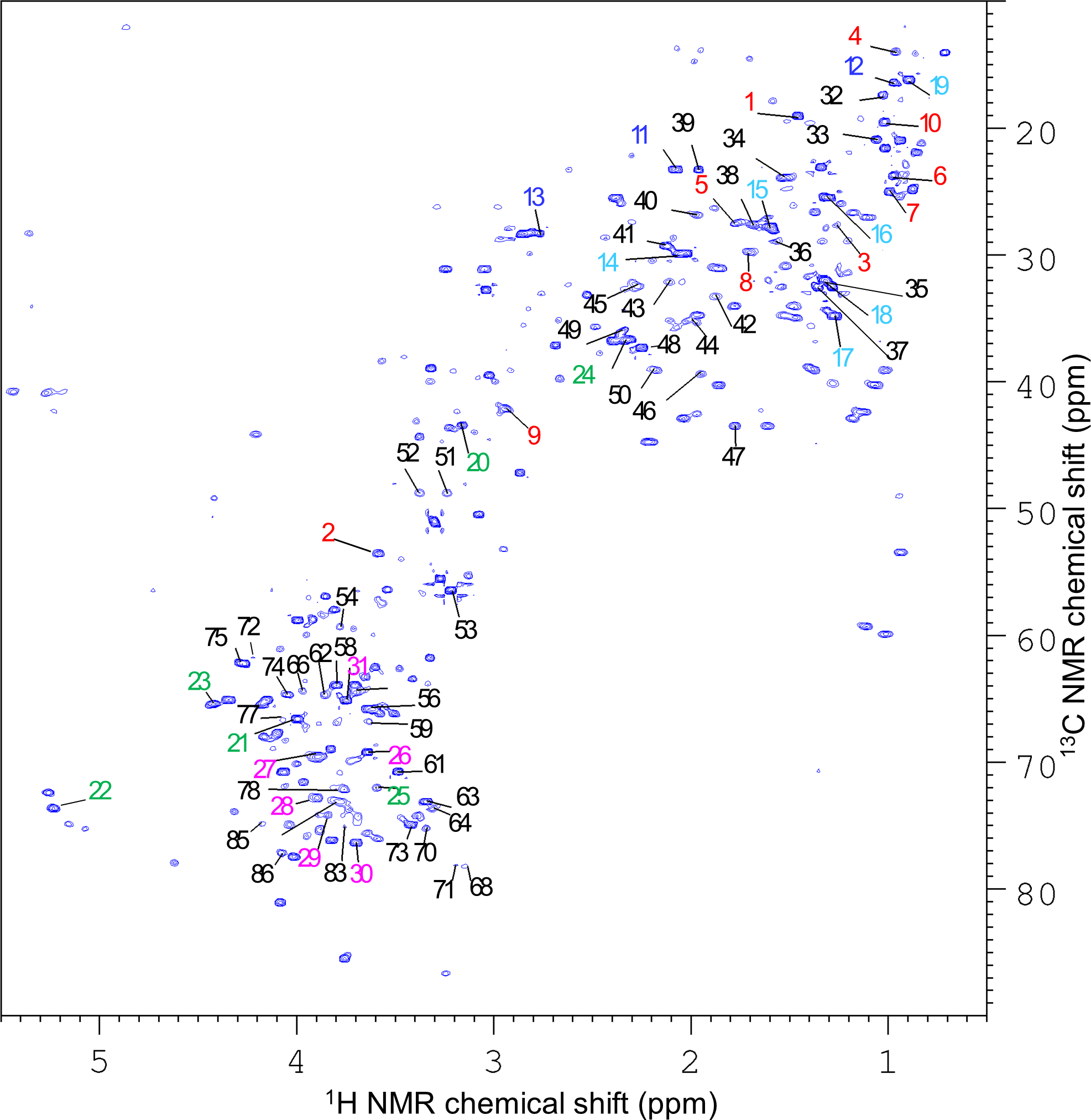

Supplement: Figure S5 — 1H–13C HSQC spectra of methanol-soluble component profiles from artificial feed. Peaks were used in Fig. 2. Red, amino acid; blue, unsaturated fatty acid; aqua, fatty acid and phospholipid. Peak numbers and annotated metabolites are listed in Supplementary Table 3. [file peerj-02-550-s005.png]

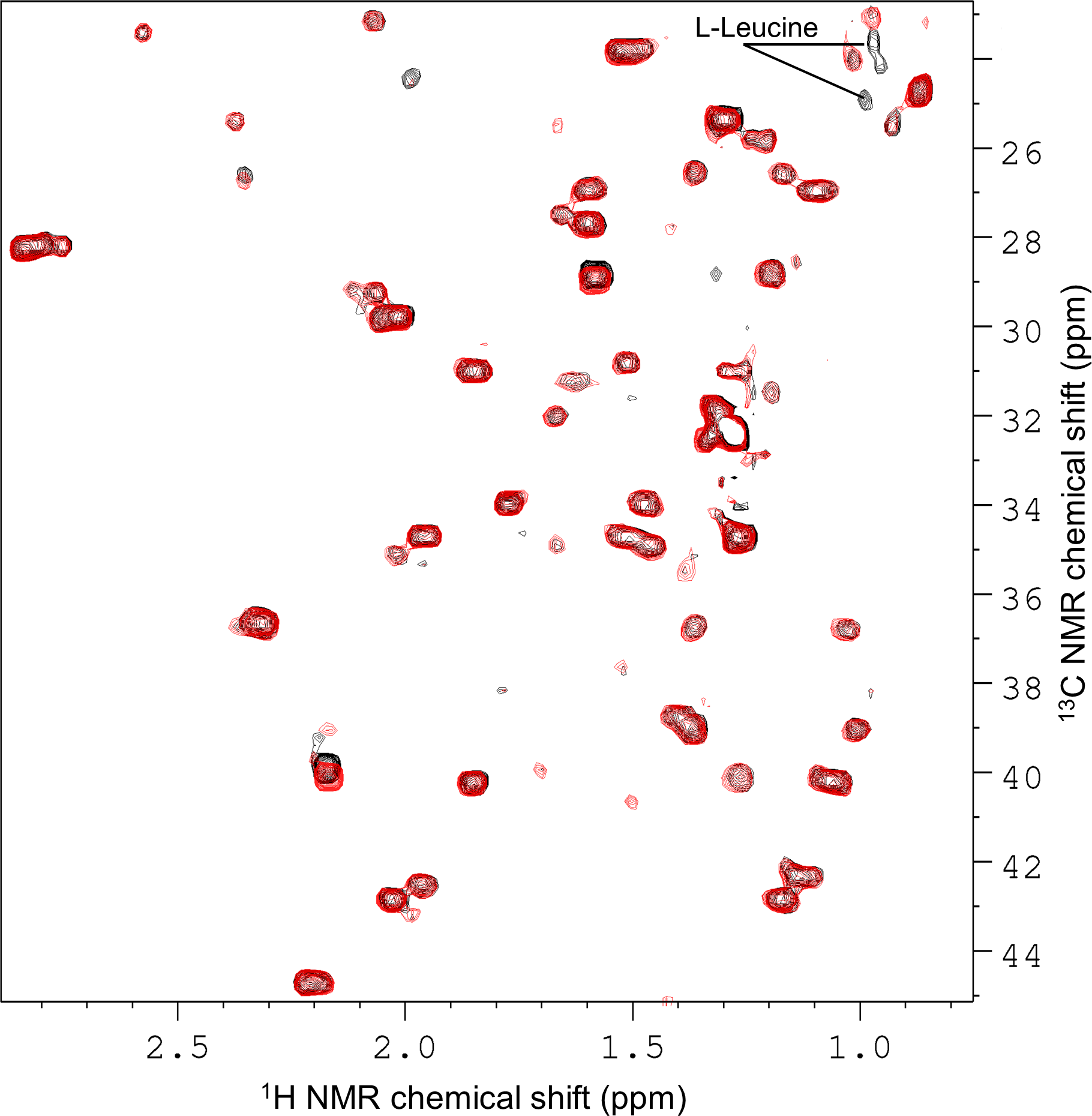

Supplement: Figure S6 — 1H–13C HSQC spectra of methanol-soluble component profiles for evaluation of retention capability of water-soluble components in fish feces of Sebastiscus marmoratus. Black and red indicate the fecal samples before and after rinsing with artificial seawater (for 15 min with shaking, 3 times), respectively. [file peerj-02-550-s006.png]

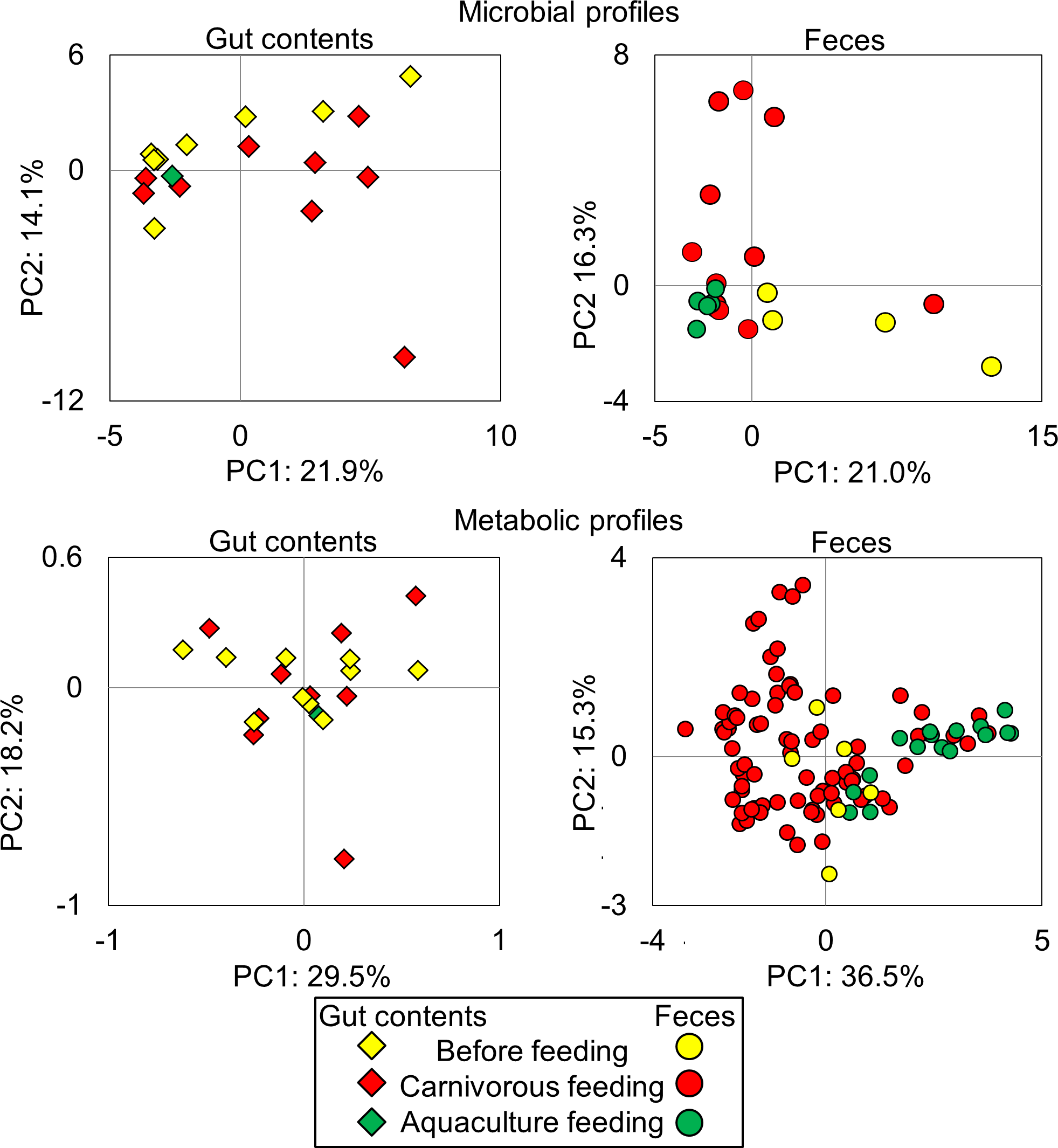

Supplement: Figure S7 — Metabolic and microbial profiles of intestinal contents and feces of 13 species of breeding fish (Table S3). PCA scores plot for microbial profile in feces (n = 22, k = 109, R2X = 0.282, R2Y = 0.241, Q2 = 0.089), microbial profile in intestinal contents (n = 19, k = 65, R2X = 0.219, R2Y = 0.141, Q2 = 0.066), metabolic profile in feces (n = 115, k = 793, R2X = 0.224, R2Y = 0.187, Q2 = 0.138), and metabolic profile in intestinal contents (n = 20, k = 786, R2X = 0.331, R2Y = 0.152, Q2 = 0.251) are shown. Triangle indicates intestinal contents, circle indicates feces. Color indicates the feeding status of breeding fish. [file peerj-02-550-s007.png]
