# Supplementary material for: Noninvasive analysis of metabolic changes following nutrient input into diverse fish species, as investigated by metabolic and microbial profiling approaches
Source: PeerJ. 2014 Oct 28;2:e550. doi: 10.7717/peerj.550 (PMC4217172; doi:10.7717/peerj.550)
Supplement: Table S1 — List of fishes for which the sequence analysis was performed. [file peerj-02-550-s008.docx]

**Supplemental Table 1**. List of fishes that performed the sequence analysis

| Scientific name | Sampling sites | Region | Habitat | H'  (Shannon–Wiener) |
| --- | --- | --- | --- | --- |
| *Paracentropogon rubripinnis* | Sagami bay, Kanagawa | Kanto | Carnivora (macrobenthos) | 4.74 |
| *Halichoeres poecilopterus* | Sagami bay, Kanagawa | Kanto | Carnivora (macrobenthos) | 5.35 |
| *Nuchequula nuchalis* | Sagami bay, Kanagawa | Kanto | Carnivora (macrobenthos) | 4.06 |
| *Pseudoblennius cottoides* | Sagami bay, Kanagawa | Kanto | Carnivora (fish) | 1.32 |
| *Scombrops boops* | Sagami bay, Kanagawa | Kanto | Carnivora (fish) | 3.82 |
| *Trachurus japonicus* | Sagami bay, Kanagawa | Kanto | Omnivora | 4.94 |
| *Hypoatherina tsurugae* | Sagami bay, Kanagawa | Kanto | Plankton-eating | 4.12 |
| *Omobranchus punctatus* | Sagami river, Kanagawa | Kanto | Omnivora | 5.99 |
| *Tridentiger bifasciatus* | Sagami river, Kanagawa | Kanto | Omnivora | 3.95 |
| *T. brevispinis* | Sagami river, Kanagawa | Kanto | Omnivora | 5.21 |
| *Apogon semilineatus* | Suruga bay, Shizuoka | Kanto | Carnivora (macrobenthos) | 3.86 |
| *Saurida elongata* | Suruga bay, Shizuoka | Kanto | Carnivora (fish) | 4.39 |
| *Parablennius yatabei* | Tokyo bay, Kanagawa | Kanto | Carnivora (macrobenthos) | 5.03 |
| *Ditrema temmincki temmincki* | Tokyo bay, Kanagawa | Kanto | Carnivora (macrobenthos) | 4.62 |
| *Sillago japonica* | Tokyo bay, Kanagawa | Kanto | Carnivora (macrobenthos) | 3.56 |
| *Sagamia geneionema* | Tokyo bay, Kanagawa | Kanto | Carnivora (macrobenthos) | 4.9 |
| *Sebastiscus marmoratus* | Tokyo bay, Kanagawa | Kanto | Carnivora (fish) | 2.86 |
| *Sebastes ventricosus* | Tokyo bay, Kanagawa | Kanto | Carnivora (fish) | 3.53 |
| *Lateolabrax japonicus* | Tokyo bay, Kanagawa | Kanto | Carnivora (fish) | 4.39 |
| *Acanthogobius flavimanus* | Tsurumi river, Kanagawa | Kanto | Omnivora | 3.68 |
| *Sardinella zunasi* | Tsurumi river, Kanagawa | Kanto | Plankton-eating | 4.27 |
| *Chelonodon patoca* | Amori river, Kagoshima | Southern | Omnivora | 2.17 |
| *Gerres equulus* | Amori river, Kagoshima | Southern | Omnivora | 5.48 |
| *Pseudolabrus eoethinus* | Anbo river, Kagoshima | Southern | Carnivora (macrobenthos) | 4.56 |
| *Periophthalmus* | East China sea, Kagoshima | Southern | Carnivora (macrobenthos) | 4.15 |
| *Lutjanus stellatus* | Kurio river, Kagoshima | Southern | Carnivora (macrobenthos) | 5.02 |
| *Lutjanus russellii* | Miyanoura river, Kagoshima | Southern | Carnivora (macrobenthos) | 4.34 |
| *Caranx sexfasciatus* | Miyanoura river, Kagoshima | Southern | Carnivora (fish) | 4.27 |
| *Terapon jarbua* | Miyanoura river, Kagoshima | Southern | Carnivora (fish) | 2.29 |
| *Acanthopagrus schlegelii* | Miyanoura river, Kagoshima | Southern | Omnivora | 2.76 |
| *Yongeichthys criniger* | Hunaura bay, Okinawa | Southern | Carnivora (macrobenthos) | 4.25 |
| *Periophthalmus argentilineatus* | Hunaura bay, Okinawa | Southern | Carnivora (macrobenthos) | 5.28 |
| *Pomadasys argenteus* | Omija river, Okinawa | Southern | Carnivora (macrobenthos) | 4.54 |
| *Rhinogobius giurinus* | Omija river, Okinawa | Southern | Omnivora | 5.4 |
| *Rhinogobius sp.* | Omija river, Okinawa | Southern | Omnivora | 5.63 |
| *Physiculus maximowiczi* | Hisanohama port, Fukushima | Tohoku | Carnivora (macrobenthos) | 1.81 |
| *Tribolodon hakonensis* | Ohisa river, Fukushima | Tohoku | Omnivora | 4.04 |
| *Hexagrammos agrammus* | Pacific sea, Fukushima | Tohoku | Carnivora (macrobenthos) | 4.73 |
| *Platichthys stellatus* | Natori river, Miyagi | Tohoku | Carnivora (macrobenthos) | 4.52 |
| *Hexagrammos otakii* | Natori river, Miyagi | Tohoku | Carnivora (macrobenthos) | 5.08 |
| *Pleuronectes yokohamae* | Natori river, Miyagi | Tohoku | Carnivora (macrobenthos) | 4.37 |
| *Mugil cephalus* | Torinoumi bay, Miyagi | Tohoku | Detritus-eating | 2.4 |
