# Supplementary material for: Noninvasive analysis of metabolic changes following nutrient input into diverse fish species, as investigated by metabolic and microbial profiling approaches
Source: PeerJ. 2014 Oct 28;2:e550. doi: 10.7717/peerj.550 (PMC4217172; doi:10.7717/peerj.550)
Supplement: Table S2 — List of fishes that performed the breeding and sampling of feces. [file peerj-02-550-s009.docx]

**Supplemental Table 2**. List of fishes that performed the breeding and sampling of feces

|  | Scientific name | Sampling Sites | Local feces | Feeds | | | Number  of individuals | Number  of  samples |
| --- | --- | --- | --- | --- | --- | --- | --- | --- |
| a | *Epinephelus septemfasciatus* | Sagami bay, Kanagawa | - | Polychaeta | Crustacea | Aquaculture feed | 3 | 33 |
| b | *Epinephelus areolatus* | Toyko bay, Kanagawa | ○ | Aquaculture feed |  |  | 1 | 3 |
| c | *Sebastiscus marmoratus* | Toyko bay, Kanagawa | - | Polychaeta & Crustacea |  |  | 15-20 | 19 |
| d | *Sebastes ventricosus* | Toyko bay, Kanagawa | - | Polychaeta |  |  | 2 | 27 |
| e | *Acanthogobius flavimanus* | Tsurumi river, Kanagawa | ○ | Polychaeta | Aquaculture feed |  | 15-20 | 23 |
| f | *Evynnis japonica* | Pacific sea, Chiba | - | Crustacea |  |  | 2 | 11 |
| g | *Sillago japonica* | Sagami bay, Kanagawa | - | Polychaeta |  |  | 6 | 5 |
| h | *Parapristipoma trilineatum* | Sagami bay, Kanagawa | ○ | Polychaeta |  |  | 1 | 3 |
| i | *Takifugu snyderi* | Pacific sea, Miyagi | ○ | Polychaeta |  |  | 1 | 7 |
| j | *Synodus ulae* | Sagami bay, Kanagawa | Only Local feces | - |  |  | 2 | 2 |
| k | *Thamnaconus modestus* | Pacific sea, Chiba | Only Local feces | - |  |  | 1 | 1 |
| l | *Trachurus japonicus* | Toyko bay, Kanagawa | Only Local feces | - |  |  | 1 | 2 |
| m | *Paralichthys olivaceus* | Pacific sea, Miyagi | Only Local feces | - |  |  | 1 | 2 |
| n | *Platycephalus sp.* | Toyko bay, Kanagawa | - | Polychaeta |  |  | 4 | 4 |
| o | *Repomucenus curvicornis* | Toyko bay, Kanagawa | - | Mosquito larva |  |  | 10 | 1 |
| p | *Achirus fasciatus* | purchased | - | Mosquito larva |  |  | 5 | 1 |
| q | *Carassius auratus auratus* | purchased | - | Goldfish feed |  |  | 50 | 1 |
| r | *Danio rerio* | purchased | - | Goldfish feed |  |  | 15 | 4 |
| s | *Eopsetta grigorjewi* | Pacific sea, Miyagi | Only Local feces | - |  |  | 1 | 1 |
| t | *Hexagrammos otakii* | Natori river, Miyagi | - | Polychaeta |  |  | 3 | 4 |
| u | *Lateolabrax japonicus* | Toyko bay, Kanagawa | - | Polychaeta |  |  | 1 | 2 |
