# Supplementary material for: Noninvasive analysis of metabolic changes following nutrient input into diverse fish species, as investigated by metabolic and microbial profiling approaches
Source: PeerJ. 2014 Oct 28;2:e550. doi: 10.7717/peerj.550 (PMC4217172; doi:10.7717/peerj.550)
Supplement: Table S3 — List of methanol-soluble compounds detected in 1H–13C HSQC spectra and annotated by SpinAssign program. [file peerj-02-550-s010.docx]

**Supplemental Table 3.** List of methanol-soluble compounds detected in ^1^H-^13^C HSQC spectra and annotated by SpinAssign program

| Peak No. | ^1^H PPM | ^13^C PPM | Type | Candidate metabolites | | | | |
| --- | --- | --- | --- | --- | --- | --- | --- | --- |
| 1 | 1.462 | 18.98 | amino acid | L-Alanine |  |  |  |  |
| 2 | 3.571 | 53.39 | amino acid | L-Alanine |  |  |  |  |
| 3 | 1.257 | 27.59 | amino acid | L-Isoleucine |  |  |  |  |
| 4 | 0.956 | 13.88 | amino acid | L-Isoleucine |  |  |  |  |
| 5 | 1.776 | 27.42 | amino acid | L-Leucine |  |  |  |  |
| 6 | 0.969 | 23.73 | amino acid | L-Leucine |  |  |  |  |
| 7 | 0.997 | 24.96 | amino acid | L-Leucine |  |  |  |  |
| 8 | 1.708 | 29.70 | amino acid | L-Lysine |  |  |  |  |
| 9 | 2.939 | 42.01 | amino acid | L-Lysine |  |  |  |  |
| 10 | 1.01 | 19.51 | amino acid | L-Valine |  |  |  |  |
| 11 | 2.077 | 23.20 | unsaturated fatty acid | Octadecatrienoic acid |  |  |  |  |
| 12 | 0.969 | 16.34 | unsaturated fatty acid | Octadecatrienoic acid |  |  |  |  |
| 13 | 2.774 | 28.30 | unsaturated fatty acid | Linoleate |  |  |  |  |
| 14 | 2.022 | 29.88 | fatty acid and phospholipid | 1-Oleoylglycerophosphocholine | Octadecenoic acid | Phosphatidylethanolamine |  |  |
| 15 | 1.598 | 27.77 | fatty acid and phospholipid | 1-Oleoylglycerophosphocholine | 1-Palmitoylglycerophosphocholine | Sebacic acid | Suberic acid | Octadecenoic acid |
|  |  |  |  | Octadecatrienoic acid | Hexadecanoic acid | Linoleate | Octadecanoic acid | Tetracosanoic acid |
|  |  |  |  | 1-Phosphatidyl-D-myo-inositol | Phosphatidylcholine | Phosphatidylethanolamine |  |  |
| 16 | 1.311 | 25.31 | fatty acid and phospholipid | 1-Oleoylglycerophosphocholine | 1-Palmitoylglycerophosphocholine | Dodecanoic acid | Linoleate | Nonanoic acid |
|  |  |  |  | Octanoic acid | Tetracosanoic acid | 1-Phosphatidyl-D-myo-inositol | Phosphatidylcholine | Phosphatidylethanolamine |
|  |  |  |  | Galactosylceramide |  |  |  |  |
| 17 | 1.27 | 34.80 | fatty acid and phospholipid | 1-Oleoylglycerophosphocholine | 1-Palmitoylglycerophosphocholine | Octadecenoic acid | Docosanoic acid | Dodecanoic acid |
|  |  |  |  | Hexadecanoic acid | Nonanoic acid | Octadecanoic acid | Tetracosanoic acid | 1-Phosphatidyl-D-myo-inositol |
|  |  |  |  | Phosphatidylcholine | Phosphatidylethanolamine | Galactosylceramide |  |  |
| 18 | 1.284 | 32.34 | fatty acid and phospholipid | Hexacosan-1-ol | Octacosan-1-ol | 1-Oleoylglycerophosphocholine | 1-Palmitoylglycerophosphocholine | Dodecanoic acid |
|  |  |  |  | Hexadecanoic acid | Octadecanoic acid | Tetracosanoic acid | 1-Phosphatidyl-D-myo-inositol | Phosphatidylcholine |
|  |  |  |  | Phosphatidylethanolamine | Galactosylceramide |  |  |  |
| 19 | 0.887 | 16.17 | fatty acid and phospholipid | 1-Oleoylglycerophosphocholine | 1-Palmitoylglycerophosphocholine | Octadecenoic acid | Docosanoic acid | Dodecanoic acid |
|  |  |  |  | Hexadecanoic acid | Linoleate | Nonanoic acid | Octadecanoic acid | Octanoic acid |
|  |  |  |  | Tetracosanoic acid | 1-Phosphatidyl-D-myo-inositol | Phosphatidylcholine | Phosphatidylethanolamine | Galactosylceramide |
| 20 | 3.157 | 43.41 | Phospholipid | Ethanolamine phosphate | 1-Phosphatidyl-D-myo-inositol | Phosphatidylethanolamine |  |  |
| 21 | 3.991 | 66.62 | Phospholipid | 1-Phosphatidyl-D-myo-inositol | Phosphatidylcholine | Phosphatidylethanolamine |  |  |
| 22 | 5.236 | 73.65 | Phospholipid | 1-Phosphatidyl-D-myo-inositol | Phosphatidylcholine | Phosphatidylethanolamine |  |  |
| 23 | 4.429 | 65.39 | Phospholipid | Phosphatidylcholine | Phosphatidylethanolamine |  |  |  |
| 24 | 2.323 | 36.56 | Phospholipid | Adipate | 1-Phosphatidyl-D-myo-inositol | Phosphatidylcholine | Phosphatidylethanolamine |  |
| 25 | 3.595 | 71.89 | Phospholipid | Galactosylceramide |  |  |  |  |
| 26 | 3.636 | 69.08 | sugar | Isomaltose | Melibiose |  |  |  |
| 27 | 3.896 | 69.43 | sugar | Panose | Raffinose |  |  |  |
| 28 | 3.896 | 72.77 | sugar | D-Galactose | Raffinose | Cellobiose |  |  |
| 29 | 3.841 | 74.00 | sugar | Raffinose |  |  |  |  |
| 30 | 3.704 | 76.29 | sugar | Melezitose | Raffinose | Panose | 1F-beta-D-Fructosylsucrose | Stachyose |
| 31 | 3.745 | 65.04 | sugar | Raffinose | 1F-beta-D-Fructosylsucrose |  |  |  |
| 32 | 1.024 | 17.40 | amino acid | L-Isoleucine |  |  |  |  |
| 33 | 1.051 | 20.91 | amino acid | L-Valine |  |  |  |  |
| 34 | 1.489 | 24.61 | amino acid | L-Lysine |  |  |  |  |
| 35 | 1.311 | 31.99 | fatty acid and phospholipid | 1-Oleoylglycerophosphocholine | 1-Palmitoylglycerophosphocholine | Sebacic acid | Octadecenoic acid | Octadecatrienoic acid |
|  |  |  |  | Docosanoic acid | Dodecanoic acid | Hexadecanoic acid | Linoleate | Nonanoic acid |
|  |  |  |  | Octadecanoic acid | Octanoic acid | Tetracosanoic acid | 1-Phosphatidyl-D-myo-inositol | Phosphatidylcholine |
|  |  |  |  | Phosphatidylethanolamine | Galactosylceramide |  |  |  |
| 36 | 1.571 | 28.82 | Phospholipid | Galactosylceramide |  |  |  |  |
| 37 | 1.352 | 32.52 | fatty acid and phospholipid | Octadecatrienoic acid | Linoleate | 1-Phosphatidyl-D-myo-inositol | Phosphatidylethanolamine |  |
| 38 | 1.681 | 27.59 | Phospholipid | Phosphatidylethanolamine |  |  |  |  |
| 39 | 1.954 | 23.20 | Phospholipid | Hexacosan-1-ol | Octacosan-1-ol | Ubiquinone-10 | 1-Phosphatidyl-D-myo-inositol |  |
| 40 | 1.968 | 26.71 | amino acid | L-Proline |  |  |  |  |
| 41 | 2.118 | 29.18 | Phospholipid | Phosphatidylethanolamine |  |  |  |  |
| 42 | 1.872 | 33.22 | amino acid | L-Lysine |  |  |  |  |
| 43 | 2.104 | 31.99 | amino acid | L-Proline |  |  |  |  |
| 44 | 2.022 | 35.15 | Phospholipid | Galactosylceramide |  |  |  |  |
| 45 | 2.269 | 32.34 | amino acid | L-Valine |  |  |  |  |
| 46 | 1.94 | 39.37 | amino acid | L-Isoleucine |  |  |  |  |
| 47 | 1.776 | 43.41 | amino acid | L-Leucine |  |  |  |  |
| 48 | 2.255 | 37.26 | unsaturated fatty acid | Suberic acid | Octadecenoic acid | Octadecatrienoic acid | Docosanoic acid | Dodecanoic acid |
|  |  |  |  | Dodecanoic acid | Hexadecanoic acid | Linoleate | Nonanoic acid | Octadecanoic acid |
|  |  |  |  | Octanoic acid | Octanoic acid | Tetracosanoic acid |  |  |
| 49 | 2.351 | 36.03 | Phospholipid | Glutarate | Phosphatidylethanolamine |  |  |  |
| 50 | 2.173 | 39.02 | Phospholipid | Galactosylceramide |  |  |  |  |
| 51 | 3.239 | 48.69 | amino acid | L-Proline |  |  |  |  |
| 52 | 3.376 | 48.69 | amino acid | L-Proline |  |  |  |  |
| 53 | 3.212 | 56.42 | Phospholipid | 1-Oleoylglycerophosphocholine | Choline | Choline phosphate | Phosphatidylcholine |  |
| 54 | 3.786 | 59.24 | amino acid | D-Cysteine |  |  |  |  |
| 55 | 3.704 | 63.81 | sugar | Lactose |  |  |  |  |
| 56 | 3.622 | 65.74 | sugar | Deoxyribose | D-Fructose | D-Tagatose |  |  |
| 57 | 3.704 | 64.51 | sugar | alpha-D-Mannose | Raffinose | 2-Deoxy-D-glucose |  |  |
| 58 | 3.8 | 63.81 | sugar | Maltotetraose | Maltose | Isomaltose | 1F-beta-D-Fructosylsucrose | Lactose |
|  |  |  |  | Panose |  |  |  |  |
| 59 | 3.636 | 66.79 | sugar | D-Sorbose |  |  |  |  |
| 60 | 3.786 | 64.51 | sugar | alpha-D-Mannose | 2-Deoxy-D-glucose | Lactulose |  |  |
| 61 | 3.486 | 70.66 | sugar | D-Allose |  |  |  |  |
| 62 | 3.855 | 64.69 | sugar | 2-Deoxy-D-glucose |  |  |  |  |
| 63 | 3.349 | 73.12 | sugar | Melezitose | Raffinose | Isomaltose | Cellobiose |  |
| 64 | 3.321 | 73.65 | sugar | alpha,alpha-Trehalose | Melezitose | Raffinose |  |  |
| 65 | 3.636 | 69.08 | Phospholipid | 1-Oleoylglycerophosphocholine | 1-Palmitoylglycerophosphocholine | Phosphatidylcholine |  |  |
| 66 | 3.978 | 64.33 | amino acid | L-Proline |  |  |  |  |
| 67 | 3.978 | 64.33 | sugar | Panose |  |  |  |  |
| 68 | 3.144 | 78.05 | sugar | D-Glucose |  |  |  |  |
| 69 | 3.677 | 69.61 | sugar | Stachyose | Deoxyribose |  |  |  |
| 70 | 3.349 | 75.06 | sugar | D-Xylose |  |  |  |  |
| 71 | 3.198 | 78.05 | Phospholipid | 1-Phosphatidyl-D-myo-inositol |  |  |  |  |
| 72 | 4.224 | 61.70 | Phospholipid | Choline phosphate |  |  |  |  |
| 73 | 3.417 | 74.88 | sugar | Maltotetraose | Maltose | 1F-beta-D-Fructosylsucrose | Panose | 6-Deoxy-D-galactose |
|  |  |  |  | 6-Deoxy-L-galactose | Isomaltose |  |  |  |
| 74 | 4.046 | 64.69 | Phospholipid | 1-Phosphatidyl-D-myo-inositol |  |  |  |  |
| 75 | 4.279 | 62.22 | Phospholipid | 1-Oleoylglycerophosphocholine | 1-Palmitoylglycerophosphocholine | Phosphatidylcholine |  |  |
| 76 | 3.786 | 70.84 | sugar | D-Fructose | Deoxyribose | beta-D-Fructose |  |  |
| 77 | 4.074 | 66.62 | Phospholipid | 1-Phosphatidyl-D-myo-inositol |  |  |  |  |
| 78 | 3.759 | 72.07 | sugar | Raffinose | Melibiose |  |  |  |
| 79 | 3.814 | 71.89 | sugar | D-Galactose | Lactulose |  |  |  |
| 80 | 3.814 | 71.89 | Phospholipid | Galactosylceramide |  |  |  |  |
| 81 | 3.773 | 73.12 | sugar | Melibiose | Stachyose | Raffinose | D-Tagatose | 6-Deoxy-D-galactose |
|  |  |  |  | 6-Deoxy-L-galactose | beta-D-Fructose |  |  |  |
| 82 | 3.759 | 75.06 | sugar | D-Altrose | Maltotetraose | 2-Deoxy-D-glucose |  |  |
| 83 | 3.759 | 75.06 | Phospholipid | 1-Phosphatidyl-D-myo-inositol |  |  |  |  |
| 84 | 3.896 | 74.18 | sugar | D-Altrose | Stachyose |  |  |  |
| 85 | 4.183 | 74.88 | Phospholipid | 1-Phosphatidyl-D-myo-inositol |  |  |  |  |
| 86 | 4.074 | 77.17 | sugar | Raffinose |  |  |  |  |
